# Increased Cholesterol Content in Gammadelta (γδ) T Lymphocytes Differentially Regulates Their Activation

**DOI:** 10.1371/journal.pone.0063746

**Published:** 2013-05-21

**Authors:** Hsin-Yuan Cheng, Runpei Wu, Abraham K. Gebre, Richard N. Hanna, Dan J. Smith, John S. Parks, Klaus Ley, Catherine C. Hedrick

**Affiliations:** 1 Division of Inflammation Biology, La Jolla Institute for Allergy & Immunology, La Jolla, California, United States of America; 2 Department of Pathology/Lipid Sciences, Wake Forest School of Medicine, Winston-Salem, North Carolina, United States of America; 3 Targeson, Inc., San Diego, California, United States of America; National Institute of Environmental Health Sciences, United States of America

## Abstract

Gammadelta (γδ) T lymphocytes respond quickly upon antigen encounter to produce a cytokine response. In this study, we sought to understand how functions of γδ T cells are differentially regulated compared to αβ T cells. We found that cholesterol, an integral component of the plasma membrane and a regulator of TCR signaling, is increased in γδ T cells compared to αβ T cells, and modulates their function. Higher levels of activation markers, and increased lipid raft content in γδ cells suggest that γδ T cells are more activated. Cholesterol depletion effectively decreased lipid raft formation and activation of γδ T cells, indicating that increased cholesterol content contributes to the hyper-activated phenotype of γδ T cells, possibly through enhanced clustering of TCR signals in lipid rafts. TCR stimulation assays and western blotting revealed that instead of a lower TCR threshold, enhanced TCR signaling through ERK1/2 activation is likely the cause for high cholesterol-induced rapid activation and proliferation in γδ T cells. Our data indicate that cholesterol metabolism is differentially regulated in γδ T cells. The high intracellular cholesterol content leads to enhanced TCR signaling and increases activation and proliferation of γδ T cells.

## Introduction

Most T cells express the αβ T cell receptor (TCR). However, a small subset of T cells expresses the γ and δ chains of the TCR. These γδ T cells represent ∼3–5% of total CD3^+^ T cells in human peripheral blood and recognize non-peptide antigens such as lipids and phosphorylated nucleotides, as well as antigens that do not require processing and presentation by MHC molecules [1], [2]. Antigen-naive γδ T cells can react quickly, within hours after pathogen infection, and thus serve an innate immunity-like role before αβ T cells and other adaptive immune responses could take place [2], [3]. A γδ T cell response is key to numerous pathogenic processes, as these cells have been shown to facilitate adaptive immune responses through various mechanisms [4]. For instance, γδ T cells promote the maturation of naïve dendritic cells during viral infection, possibly through the production of proinflammatory cytokines such as TNFα, IFNγ, and IL-6 [5]. γδ T cells are also shown to induce robust CD8^+^ T cell responses by cross-presenting microbial and tumor antigens to CD8^+^ T cells [6].

Several groups have investigated unique gene expression patterns of γδ T cells upon stimulation as hallmarks to distinguish them from αβ T cells, but have reported finding relatively similar expression profiles thus far [7], [8], [9]. One of the most noteworthy findings was by Fahrer et al., who reported that γδ and αβ T cells show distinct expression patterns of both lipid metabolism and inflammatory genes upon *Y. pseudotuberculosis* infection [10]. These investigators reported that mRNA for several lipid metabolism genes were expressed only in the γδ T cell samples. Another recent study reported that the response of γδ T cells toward influenza virus was potently inhibited by blocking HMG-CoA reductase, the rate-limiting enzyme in cholesterol biosynthesis, suggesting sterol metabolism may be important for the function of γδ T cells [11].

Cholesterol maintains proper permeability and fluidity of the mammalian cell membrane to ensure cell growth and function. Cholesterol plays a role in mediating signal transduction by assisting the formation of lipid rafts, the specialized microdomains for organizing signaling molecules [12]. However, cholesterol levels must be properly regulated as excess sterol results in adverse effects on normal cell functions as well as the development of diseases such as atherosclerosis. Several studies have demonstrated that the homeostasis and functions of various T cell subsets are strongly linked to cellular and environmental cholesterol levels. Resting peripheral CD4^+^ T cells and the Th1 responses were both increased after *in vivo* cholesterol enrichment [13]. Coincidentally, we also reported that CD4^+^ T cells had increased intracellular cholesterol content and proliferative advantage in the absence of ABCG1, an cholesterol efflux transporter [14]. On the other hand, proliferation of NKT cells in response to αGalCer stimulation was lower in hypercholesterolemic ApoE^−/−^ mice [15]. In this report, we provide novel evidence by which αβ and γδ T cells are differentially regulated by intracellular cholesterol content. We found that intracellular cholesterol levels are basally elevated in γδ T cells and that this contributes to their “primed for action” phenotype by favoring TCR clustering and signaling.

## Methods

### Mice

C57BL/6J (000664) mice were purchased from The Jackson Laboratory. Mice were fed a standard rodent chow diet and housed in microisolator cages in a pathogen-free facility. All experiments followed guidelines of the La Jolla Institute for Allergy and Immunology Animal Care and Use Committee, and approval for use of rodents was obtained from the La Jolla Institute for Allergy and Immunology according to criteria outlined in the Guide for the Care and Use of Laboratory Animals from the National Institutes of Health. Mice were euthanized by CO_2_ inhalation.

### Flow Cytometry

Spleens or peripheral lymph nodes (cervical, axillary, brachial, and inguinal) were excised and pushed through a 70-µm strainer. Red blood cells were lysed in RBC Lysis Buffer according to the manufacturer's protocol (BioLegend). All samples were collected in Dulbecco's PBS (Gibco) with 2 mM EDTA and were stored on ice during staining and analysis. 2–6×10^6^ cells were resuspended in 100 µl flow staining buffer [1% FBS plus 0.1% NaN_3_ in PBS]. Fc receptors were blocked for 10 min and surface antigens were stained for 30 min at 4°C and washed twice with flow staining buffer. LIVE/DEAD Yellow Fixable Dead Cell Stain (Invitrogen) was used for analysis of viability, and forward- and side-scatter parameters were used for exclusion of doublets from analysis.

For measurement of intracellular lipids, after surface markers staining, cells were stained for an additional 15 min at room temperature with 5 ng/µl Nile Red (Enzo) and then washed twice.

For measurement of lipid rafts, cells were stained with Alexa Fluor 488-conjugated Cholera toxin subunit B (1/1000 dilution; Molecular Probes) together with other surface staining antibodies for 30 min at 4°C, and then washed twice.

Cellular fluorescence was assessed using LSR II flow cytometer (BD Biosciences) and percentages of subsets and median fluorescence intensity (MFI) were analyzed with FlowJo software (TreeStar version 9.2).

Fluorescence-labeled antibodies used were- anti-CD3 (145-2C11, eBioscience), anti-TCRβ (H57-597, eBioscience), anti-γδ TCR (eBioGL3, eBioscience), anti-CD69 (H1.2F3, BD Pharmingen), anti-CD44 (IM7, eBioscience), and anti-CD62L (MEL-14, eBiocience).

### *In vivo* proliferation assay

C57BL/6J mice were intraperitoneally injected with 1 mg BrdU (BD Pharmingen). Spleens were harvested after 3 days, and BrdU incorporation was detected following manufacturer's instruction (FITC BrdU Flow Kit, BD Pharmingen).

### Quantification of cholesterol content

Splenocytes were isolated from C57BL/6J mice and stained with anti-CD3, -TCRβ, -γδ, and LIVE/DEAD Yellow Fixable Dead Cell Stain (Invitrogen). 3 spleens were pooled per sample. Stained cells were sorted into αβ (live, CD3^+^, TCRβ^+^, and γδ^−^) and γδ (live, CD3^+^, γδ^+^, and TCRβ^−^) T cells by FACSAria flow cytometer (BD Biosciences). ∼9×10^6^ αβ T cells and ∼1.5–2×10^5^ γδ T cells were collected per mouse sample. 6×10^5^ αβ and all of the γδ T cells (∼1.5–2×10^5^) were used for GC quantification. After several washes with PBS, the cell pellet was extracted with isopropanol containing 5-cholestane as internal standard. Total and free cholesterol content was determined by gas–liquid chromatography and normalized to cell numbers, as previously described [16]. Cholesteryl ester was calculated as (total cholesterol - free cholesterol) ×1.67. Multiplying by 1.67 corrects for the average fatty acid mass that is lost during saponification.

### Quantification of cell size

αβ and γδ T cells were isolated by cell sorting as described in the quantification of cholesterol content. Diameters of single cells were measured by Multisizer IV Coulter Counter (Beckman Coulter).

### PCR array

Splenocytes were isolated from C57BL/6J mice. Untouched T cells were purified by Pan T Cell Isolation Kit II (Miltenyi Biotec), and were then separated into αβ and γδ T cells by TCRγ/δ^+^ T Cell Isolation Kit (Miltenyi Biotec). Spleens from 6 mice were pooled per sample. The purity of cells was assessed by flow cytometry and was above 90%. Total RNA was collected with the RNeasy Plus Micro Kit according to the manufacturer's protocol (Qiagen). RNA purity and quantity was measured with a nanodrop spectrophotometer (Thermo Scientific). 150 ng RNA was used to for cDNA synthesis with RT^2^ First Strand cDNA Kit (SABiosciences). Gene expression was detected using Lipoprotein Signaling & Cholesterol Metabolism PCR Array (PAMM-080, SABiosciences). Relative gene expression was analyzed using ΔΔC_t_ based fold-change calculations. Expression level of αβ T cells was set as 1.

### Quantitative RT-PCR

Magnetic beads purified αβ and γδ T cells from C57BL/6J splenocytes and total RNA extraction were stated above. Spleens from 3–4 mice were pooled per sample. 100 ng RNA was used for cDNA synthesis with iScript cDNA Synthesis Kit (Bio-Rad). mRNA expression was measured in real-time quantitative PCR using the following predesigned TaqMan gene expression assays (Applied Biosystems): *Abca1* (Mm01350760_m1), *Pcsk9* (Mm01263610_m1), *Acat1* (Mm00486279_m1), *Acat2* (Mm00448823_m1), *Lcat* (Mm00500505_m1), *Trerf1* (Mm00553936_m1). Samples were assayed on a LightCycler 480 Real-Time PCR System (Roche). Threshold cycles (CT) were determined by an in-program algorithm assigning a fluorescence baseline based on readings prior to exponential amplification. Fold change in expression was calculated using the ΔΔC_t_ method with *Gapdh* as an endogenous control. Expression level of αβ T cells was set as 1.

### Confocal microscopy imaging

αβ and γδ T cells were isolated from female C57BL/6J mice using magnetic beads as stated in the PCR array section above. Cells were stained with Alexa Fluor 488-conjugated Cholera toxin subunit B (1/1000 dilution; Molecular Probes), washed twice with PBS, and spun unto glass slides. Slides were imaged with a FluoView FV10i confocal laser-scanning microscope (Olympus).

### *In vitro* cholesterol loading and depletion

C57BL/6J splenocytes were stimulated with Dynabeads mouse T-activator CD3/CD28 beads (Invitrogen) at 1 bead: 1 cell ratio in the absence or presence of 1 mM methyl β-cyclodextrin (Sigma-Aldrich) to deplete cholesterol, or 10 or 20 µg/ml cholesterol-loaded cyclodextrin (Sigma-Aldrich) to load cells with cholesterol for 2 or 4 hours. Cells were then removed from beads, stained for lipid rafts and activation markers, and analyzed by flow cytometry as described above.

### *In vitro* T cell stimulation and proliferation assay

Splenocytes isolated from C57BL/6J mice were stained with 2.5 µM CFSE (Invitrogen) for 10 min at 37°C, and washed extensively. Cells were then plated at 5×10^5^ cells/well in a 96-well U-bottomed plate pre-coated with various concentrations of αCD3 antibody (BD Pharmingen). Soluble αCD28 antibody (BD Pharmingen) was added at 1 µg/ml. 24 hours later, cells were stained with surface markers, and CFSE dilution in αβ and γδ subsets was determined by flow cytometry. In some experiments, U0126 (Sigma-Aldrich) was added to cultures at 10 and 25 µM to inhibit ERK1/2 phosphorylation.

### Western Blotting

C57BL/6J splenocytes were sorted into αβ (live, CD3^+^, TCRβ^+^, and γδ^−^) and γδ (live, CD3^+^, γδ^+^, and TCRβ^−^) T cells. Spleens of 25 mice were pooled for sorting. All of the sorted γδ T cells (4.3×10^6^) and same number of αβ T cells were divided into 2 equal portions for generation of both unactivated and activated samples. Cells were either left untreated or incubated with 20 µg/ml αCD3 and αCD28 antibodies for 5 min at 37°C, and then cross-linked by 20 µg/ml goat anti-hamster IgG (Invitrogen) for another 2 min at 37°C. Cells were then lysed with RIPA buffer (50 mM Tris, pH 7.4, 150 mM NaCl, 1 mM EDTA, 1 mM EGTA, 1 mM NaVO_4_, 1 mM NaF, 0.5% NP40, 0.1% Brij35, 0.1% deoxycholic acid) and ultrasonication. Protein quantification was performed using BCA Protein Assay Reagent (Thermo Scientific). 30 µg protein per sample was loaded into SDS-PAGE, and sequentially immunoblotted with the following antibodies- anti-phospho-ERK1/2 (1∶250; Cell Signaling #4370), anti-ERK1/2 (1∶1000; Cell Signaling #4695), and anti-β Tubulin (1∶250; Cell Signaling #2128).

### Statistical analysis

All results are expressed as mean ± SEM. Results were analyzed by Student *t* test or ANOVA. Unpaired Student's *t* test and one-way analysis of variance were used for comparison of experimental groups. Statistical analysis is performed using GraphPad Prism software version 5.0b (GraphPad Software, Inc.). A probability value of less than 0.05 is considered significant.

## Results

### Cholesterol metabolism-related genes are differentially regulated in αβ and γδ T cells

**Figure 1:**
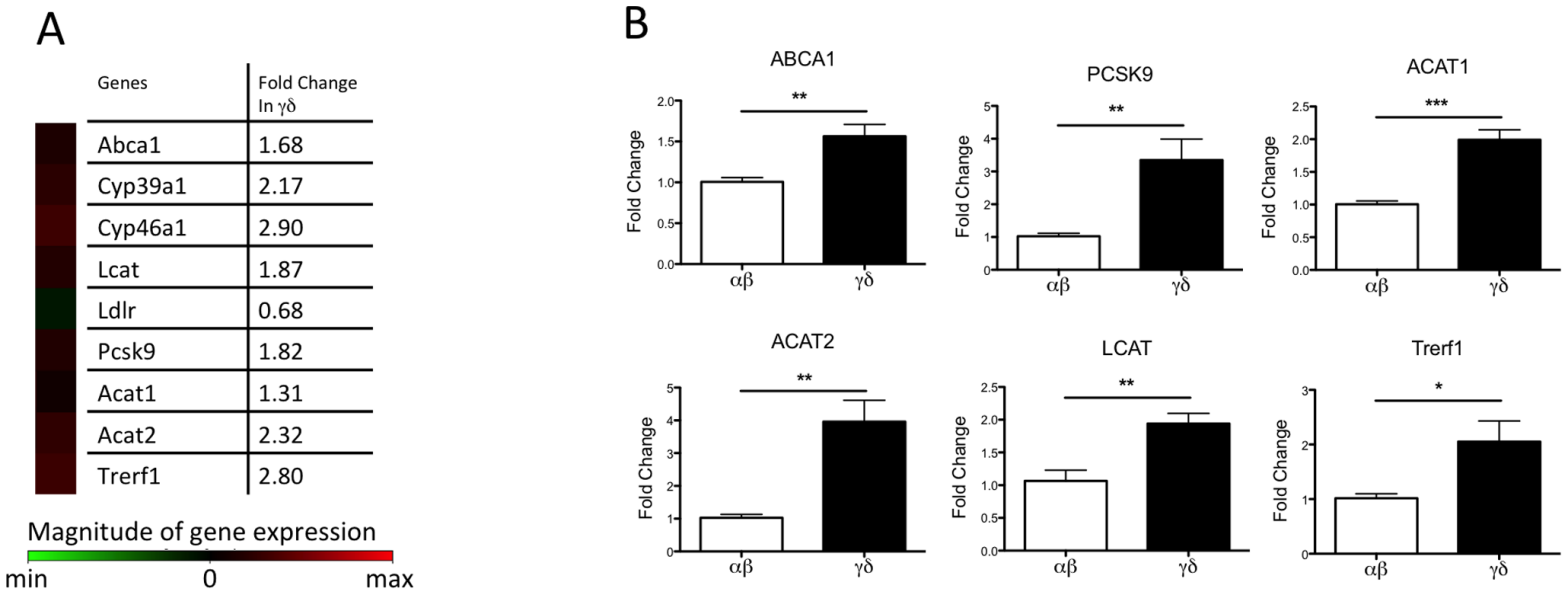
Cholesterol metabolism is differentially regulated in αβ and γδ T cells. (A) Heat map of sterol PCR array. Genes involved in sterol esterification, utilization, and efflux are upregulated in γδ T cells. Gene expression of αβ T cells was used as control to calculate fold change and hence not shown in the heat map. αβ and γδ T cells were isolated from spleens of the same female C57BL/6J mice by immuno-magnetic beads. (B) Quantitative RT-PCR analysis of genes regulating sterol metabolism. mRNA was isolated from αβ and γδ T cells from the same female C57BL/6J mice, and gene expression was detected by Taqman-based assays. *Gapdh* was used to normalize gene expression, and gene expression in αβ T cells were set as 1. N = 6. One sample was pooled from 3 mouse spleens. Results were shown in mean ± SEM. * P<0.05, ** P<0.01, *** P<0.001.

Cholesterol is essential for maintaining proper permeability and fluidity in the cell membrane, and its level is tightly regulated within the cell. T cell proliferation is strongly linked to cellular cholesterol level and the regulation of genes involved in cholesterol metabolism [17]. To investigate whether cholesterol metabolism is differentially regulated in αβ and γδ T cell subsets, we examined the expression profiles of cholesterol-related pathway genes in αβ and γδ T cells from C57BL/6J mice with a pathway-based microarray. The result showed that many genes involved in esterification (*Acat1*, *2* and *Lcat*) or utilization (*Cyp39a1*, *Cyp46a1*, *Trerf1*) of free cholesterol were upregulated in γδ T cells. The expressions of *Pcsk9*, which downregulates LDLR posttranslationally [18], and *Abca1*, the main efflux transporter for cholesterol, were up-regulated in γδ T cells as well (Fig. 1A). Genes relatively abundant and with significant fold-changes were confirmed in Taqman-based quantitative RT-PCR assays. Consistent with the PCR array results, the γδ T cells had a 1.6 fold increase in *Abca1*, a 3.4 fold increase in *Pcsk9*, a 2 fold increase in *Acat1*, a 4 fold increase in *Acat2*, a 2 fold increase in *Lcat*, and a 2.1 fold increase in *Trerf1*, compared to αβ T cells (Fig. 1B). These array data suggest that γδ T cells express effort to lower free intracellular cholesterol levels by up-regulating genes involving in sterol efflux, esterification or other utilization pathways, possibly in response to higher intracellular cholesterol content.

### Cholesterol content and lipid rafts are differentially regulated in αβ and γδ T cells

**Figure 2:**
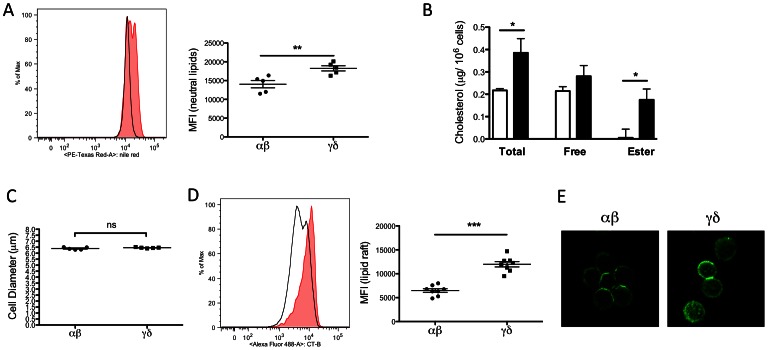
γδ T cells have increased intracellular neutral lipids, cholesterol, and lipid rafts. (A) Splenocytes were isolated from C57BL/6J mice. Intracellular lipid was quantified by Nile Red staining. Left: A representative plot shows the relative intensity of Nile Red staining on αβ (solid black line) and γδ (tinted shade) T cells. Right: Graph shows the median florescent intensity of Nile Red staining on αβ and γδ T cells. Results were averaged from 5 mice, and shown in mean ± SEM. (B) Total and free cholesterol was quantified in purified αβ (white) and γδ (black) T cells from C57BL/6J mouse spleens by gas chromatography. Cholesteryl ester was calculated as the difference between total and free cholesterol (multiplied by 1.67). Intracellular total cholesterol and cholesteryl ester content is significantly higher in γδ T cells. Cholesterol level was normalized to cell numbers. N = 5. (C) αβ and γδ T cells were isolated from splenocytes of C57BL/6J mice. Average cell diameters were obtained from a Multisizer IV Coulter Counter (Beckman Coulter) and showed no significant difference (ns) in αβ and γδ T cells. Results were averaged from 5 mice, and shown in mean ± SEM. (D) Staining for GM1 shows significantly higher lipid raft content on the plasma membrane of γδ T cells. Splenocytes were isolated from C57BL/6J mice. GM1 of lipid raft was identified by cholera toxin subunit B (CT-B) binding. Left: A representative plot shows the relative intensity of CT-B staining on αβ (solid black line) and γδ (tinted shade) T cells. Right: Graph shows the median florescent intensity of CT-B staining on αβ and γδ T cells. Results were averaged from 8 mice. Results were shown in mean ± SEM. * P<0.05, ** P<0.01, *** P<0.001. (E) Confocal microscopic images show increased lipid rafts staining on the membrane of γδ T cells (300×). Green: GM1.

To investigate whether lipid and cholesterol levels are different in αβ and γδ T cells, we used Nile Red as a probe to detect neutral lipids (consisting mostly of cholesteryl ester and triglyceride [19], [20]). γδ T cells showed a significant increase in Nile Red staining compared to αβ T cells, indicating that γδ T cells contained more neutral lipid (Fig. 2A). We next specifically measured cholesterol levels in the two subsets. Resting αβ and γδ T cells were purified from C57BL/6J mice, and the intracellular cholesterol content in each subset was measured by gas chromatography. Interestingly, we found that the total intracellular cholesterol content was significantly higher in γδ T cells (0.39±0.03 vs 0.22±0.00 µg/10^6^ cells in αβ T cells, P<0.05). Levels of free cholesterol seemed higher in the γδ T cells, but the difference did not reach statistical significance. Cholesteryl ester levels were also higher in γδ T cells (0.18±0.05 vs 0.01±0.04 µg/10^6^ cells in αβ T cells, P<0.05) (Fig. 2B). The increase in cholesteryl ester content correlates with the increase in intracellular neutral lipids observed by Nile Red staining of γδ T cells. Average cell diameter appeared to be the same for αβ and γδ T cells (6.39±0.05 vs 6.45±0.02 µm in αβ T cells, P = 0.222), hence the increased intracellular cholesterol level was not due to a larger cell size of γδ T cells (Fig. 2C).

Cholesterol is an indispensible component of membrane lipid rafts, which modulate TCR signaling by clustering proteins involved in TCR signaling. Perturbation of lipid rafts has been shown to interfere with T cell activation [21], [22], [23]. To learn whether the increase in cholesterol levels in γδ T cells impacted their membrane lipid raft content, fluorescently-labeled CT-B, which binds to an essential raft-associated protein, GM1, was used to quantify lipid rafts on the plasma membrane [14], [24]. Approximately 2-fold more CT-B bound to the plasma membrane of γδ compared to αβ cells, suggesting an increase in plasma membrane lipid raft content in γδ cells (Fig. 2D). Microscopic images of CT-B staining also clearly showed more aggregation of lipid rafts on the plasma membranes of γδ cells (Fig. 2E). Aggregation of lipid rafts and raft-associated proteins is positively associated with TCR signaling and T cell activation [23]. Taken together, these data suggest that higher cholesterol content in γδ cells results in increased membrane lipid raft content, which could in turn, change TCR signaling in the γδ T cells.

### Cholesterol depletion reduces the activated phenotype of γδ T cells

**Figure 3:**
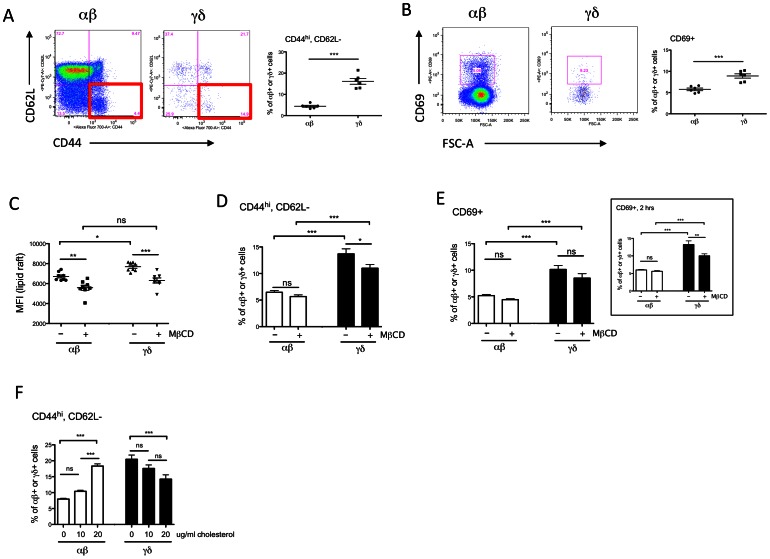
γδ T cells are more activated *in vivo*. Cholesterol depletion reduces the activated phenotype of γδ T cells. Cholesterol addition significantly increases the activation status of αβ T cells. (A–B) Staining of T cell activation markers shows more γδ T cell are in the activated state *in vivo*, comparing to αβ T cells. Splenocytes were isolated from C57BL/6J mice. T cell activation was identified by (A) CD44^hi^ CD62L^−^ and (B) CD69^+^ cells. Representative flow cytometric plots are shown on the left and the percentages of activated T cells are graphed on the right. Results were averaged from 6 mice, and shown in mean ± SEM. (C–E) Splenocytes from C57BL/6J were stimulated with T-activator CD3/CD28 beads for 4 hours in the presence or absence of MβCD to remove cholesterol from the plasma membrane. (C) Reduction of lipid raft levels in αβ and γδ T cells with cholesterol depletion. The reduction was more significant in γδ T cells, and the difference in lipid raft level became insignificant in αβ and γδ T cells after MβCD treatment. Lipid raft content was expressed in median florescent intensity of CT-B staining. (D) The percentage of CD44^hi^ CD62L^−^ cells was significantly lower in γδ T cells after 4 hours of cholesterol depletion. (E) The difference in expression of early activation marker CD69 was not statistically significant in both αβ or γδ T cells after MβCD treatment for 4 hours. However, the reduction of CD69 was significant in γδ T cells at 2 hours (inset) Results were averaged from 8 mice. Results were shown in mean ± SEM. (F) C57BL/6J splenocytes were stimulated with T-activator CD3/CD28 beads in the presence or absence of 10 and 20 µg/ml cholesterol for 4 hours. The percentage of CD44^hi^CD62L^−^ cells was significantly increased in αβ T cells in a dose-dependent manner of cholesterol addition. On the other hand, activated γδ T cells were reduced with excess cholesterol. Results were averaged from 8 mice and shown in mean ± SEM. * P<0.05; ** P<0.01; *** P<0.001; ns not statistically significant.

To confirm that the increased cholesterol content of γδ cells results in enrichment of membrane lipid rafts, which in turn would likely impact TCR signaling and activation, we manipulated cholesterol levels in αβ and γδ T cells and examined their lipid raft content and activation status *in vitro*. Examination of surface markers shows γδ T cells possess an activated status *in vivo* (increased percentages of CD44^hi^ CD62L^−^ and CD69^+^ cells) (Fig. 3A–B). The enhanced activation of γδ T cells again suggests that these cells are more “primed for action” at baseline or resting. αβ and γδ T cells from C57BL/6J spleen were stimulated with αCD3/CD28 antibodies in the absence or presence of methyl β-cyclodextrin (MβCD) to remove cellular cholesterol [25]. Lipid raft content and T cells activation markers were assessed by flow cytometry. Compared to αβ T cells, γδ T cells have a higher lipid raft content in the absence of MβCD (P<0.05). In cells treated with MβCD, the lipid raft content was lowered to similar levels in both αβ and γδ T cells (Fig. 3C). After incubation with MβCD for 4 hours, we observed a reduction in percentages of CD44^hi^CD62L^−^ γδ cells, but not αβ cells (Fig. 3D). With 4 hours of MβCD treatment, we found a slight but not significant reduction of early T cell activation marker CD69 (Fig. 3E). However, at 2 hours of cholesterol depletion, CD69^+^ cells were significantly lowered in only the γδ cells (P<0.01) (Fig. 3E, inset). We reasoned that since CD69 is one of the earliest expressed markers in T cell activation [26], its expression pattern may reflect T cell activation status early upon stimulation. We also used cholesterol-loaded cyclodextrin to enhance cellular cholesterol content in αβ and γδ T cells [27], [28]. Interestingly, we found that the percentage of CD44^hi^CD62L^−^ cells was significantly increased in αβ T cells in a dose-dependent manner with cholesterol addition. On the other hand, activated γδ T cells were gradually reduced with excess cholesterol, possibly due to oversaturation of membrane cholesterol (Fig. 3F). The fact that αβ T cells, which had less cellular cholesterol at baseline, became activated with cholesterol loading supports the idea that increased cholesterol content leads to the primed phenotype in T cells.

### γδ T cells are more proliferative and show enhanced basal ERK1/2 phosphorylation

**Figure 4:**
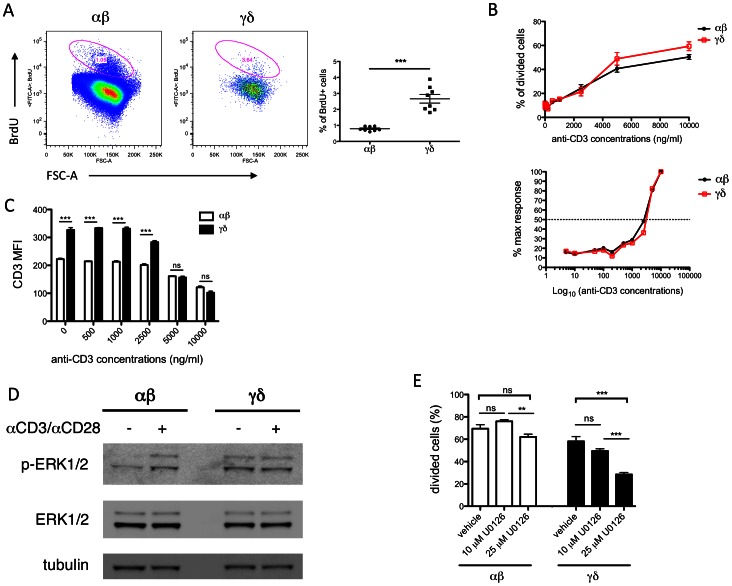
γδ T cells have similar TCR threshold but enhanced ERK1/2 phosphorylation comparing to αβ T cells. (A) Percentages of BrdU^+^ proliferative cells are higher in the γδ T cell subset *in vivo*. Left: representative plots of BrdU^+^ populations (circled) in αβ and γδ T cells. Right: Graph of percentages of BrdU^+^ cells in αβ and γδ T cells. C57BL/6J mice received single dose of BrdU (1 mg) via intraperitoneal injection, and splenocytes were isolated 3 days later. BrdU^+^ cells were identified by fluorescently labeled anti-BrdU antibody and flow cytometry. Results were averaged from 8 mice, and shown in mean ± SEM. *** P<0.001. (B) Top: Splenocytes from C57BL/6J mice were labeled with CFSE (2.5 µM), and then stimulated with 0–10 µg/ml plate-bound αCD3 and 1 µg/ml soluble αCD28 for 24 hours. CFSE content in cells was measured by flow cytometry and graphed against αCD3 concentrations. Results were averaged from 5 mice, and shown in mean ± SEM. Lower: Log scaling of αCD3 concentration against % of maximum response was used to calculate EC_50_. (C) CD3 expression in γδ T cells was higher than in αβ T cells under no or lower stimulation, but decreased more dramatically with stronger stimulation. The experimental condition was the same as in (B). (D) Western blotting shows high levels of phosphorylated ERK1/2 in γδ T cells, relative to αβ T cells, at baseline. αβ and γδ T cells were isolated from the same set of C57BL/6J mice via FACS sorting. Cells were either untreated, or incubated with αCD3 and αCD28 antibodies (20 µg/ml each) for 5 minutes and then with anti-hamster IgG (20 µg/ml) for additional 2 minutes at 37°C. Cells were lyzed, separated by SDS-PAGE, and immunoblotted with indicated antibodies. The image was representative of 3 independent experiments. (E) Inhibition of ERK1/2 significantly lowers proliferation in γδ T cells *in vitro*. Splenocytes from C57BL/6J mice were labeled with CFSE (2.5 µM), and then stimulated with 1 µg/ml plate-bound αCD3 and 1 µg/ml soluble αCD28 for 24 hours, in the absence (vehicle) or presence of 10 µM and 25 µM U0126. CFSE dilution in cells was measured by flow cytometry, and used to calculate cell proliferation. Results were averaged from 7–8 mice, and shown in mean ± SEM. ** P<0.01; *** P<0.001; ns not statistically significant.

γδ T cells play a critical role in the initial stages of various pathophysiological conditions. Compared to conventional αβ T cells, γδ T cells respond much faster when faced with microbial and tumor invasions [4], [29]. Indeed, we found a significant increase in BrdU incorporation in γδ T cells (2.67±0.09% of total γδ^+^) from C57BL/6J mouse spleen compared to αβ T cells (0.79±0.02% of total αβ^f+^, P<0.001) (Fig. 4A). Together with the increased expression of activation markers (Fig. 3A–B), our data suggesting that γδ T cells possess a more “primed for action” status at baseline in the absence of any external stimulation.

Next, we wanted to investigate the molecular mechanisms by which cholesterol stimulates γδ T cell activation. Because the accumulated cholesterol favors lipid raft formation, which could modulate TCR signaling, we first considered the possibility that γδ T cells had a lower TCR threshold [23]. To examine this hypothesis, we stimulated resting αβ and γδ T cells with a fixed concentration of soluble αCD28 and an increasing concentration of plate-bound αCD3 antibodies, and measured proliferation using CFSE dilution [14]. We found that γδ T cells are more proliferative at the higher αCD3 concentration (Fig. 4B, top). However, when the log scaling of αCD3 concentration versus percentage of maximum response was plotted to determine the EC_50_ values, we did not see a significant difference in TCR threshold between αβ and γδ T cells (Fig. 4B, lower). Our data sugest that an enhanced TCR signaling strength, but not a lower TCR threshold, likely accounts for the cholesterol-induced activation and proliferation in γδ T cells. We also noticed that with no or lower levels of αCD3 stimulation, membrane CD3 expression was significantly higher in γδ T cells compared to αβ T cells. This again indicates a stronger TCR signaling in the γδ T cells basally or with low levels of stimulation. However, the expression of CD3 was downregulated and gradually became indifferent in both αβ and γδ T cells under stronger stimulation (Fig. 4C)

To determine whether basal TCR signaling is indeed increased in the γδ T cells, we stimulated purified resting αβ and γδ T cells with αCD3 and αCD28 antibodies, and examined the phosphorylation status of ERK1/2, a well-studied TCR downstream effector for proliferation [30], [31]. We found an increase in basal ERK1/2 phosphorylation in unstimulated γδ T cells, compared to almost no basal phosphorylation in αβ T cells (Fig. 4D). The enhanced ERK1/2 phosphorylation likely accounts for the increased proliferation and activation of γδ T cells in the resting status (Fig. 3A–B, 4A).

Enhanced TCR signaling and ERK1/2 phosphorylation lead to T cell hyperplasia. To confirm that enhanced ERK1/2 signaling accounts, at least in part, for the hyperproliferative phenotype of γδ T cells, we performed an *in vitro* proliferation assay in the presence of an ERK1/2 inhibitor, U0126. Indeed, U0126 incubation significantly inhibited γδ T cell proliferation in a dose-dependent manner. However, under the same condition, the overall growth inhibition effect was not significant in the αβ T cells (Fig. 4E). The results provide further evidence that enhanced ERK1/2 signaling contributes to the hyperproliferative phenotype of γδ T cells.

## Discussion

αβ and γδ T cells share many common features, yet are functionally and metabolically distinct. For example, expression of activation markers, cytokines, and TCR signaling pathways are very similar in the two subsets [32], [33], [34]. However, γδ T cells are negative for both CD4 and CD8, and are MHC-independent in antigen recognition. Their rapid responses upon antigen encounter also distinguish them from αβ T cells. γδ cells are thought to be involved in innate immunity whereas αβ cells are involved in adaptive immunity [2], [3], [4]. To date, very little is known about how antigen-naïve γδ T cells hold the “primed for action” status and are able to become activated and proliferative in such a short period of time. In this report, we provide novel insights for the distinct phenotypes and differential regulation of αβ and γδ T cells. We showed that, in accordance with previous reports [35], [36], γδ T cells proliferate faster and are more activated than αβ T cells at resting conditions (Fig. 3A–B and 4A). We found that γδ T cells have significantly higher intracellular neutral lipid and cholesterol content (Fig. 2A–B). Furthermore, we found that the increased cholesterol content in γδ T cells results in an increase in lipid raft formation (Fig. 2D–E), which favors TCR clustering and signaling. The enhanced TCR signaling is demonstrated by elevated ERK1/2 phosphorylation, proliferation, and activation marker expression in γδ T cells (Fig. 3A–B, 4A, D). Thus, γδ T cells are kept in a “primed for action” status at a resting state, and are ready to undergo rapid proliferation and activation. We show that cholesterol, at least in part, modulates their function.

One of the best-studied functions of lipid rafts is the regulation of TCR signaling. By clustering TCR molecules and their associated factors, lipid rafts provide a concentrated and stable signaling micro-domain for TCR-dependent T cell activation [22], [23], [37]. Cholesterol, a key component of lipid rafts, is indispensible for TCR-dependent functions, and the modulation of cholesterol levels tightly correlates with the strength of TCR signaling. For example, cholesterol depletion of lymphocytes results in reduced raft domains and subsequent dysregulated T cell signaling [38]. On the other hand, a recent study showed that enriching membrane cholesterol by squalene enhances the responses of CD4^+^ T cells *in vivo* [13]. The hyper-activation of T cells in the autoimmune disease systemic lupus erythematosus is likely caused by high cholesterol-induced lowering of the TCR threshold [21]. Due to the increase in cholesterol content in γδ cells, we initially hypothesized that their hyper-active phenotype was caused by a lowered TCR threshold [23]. However, to our surprise, we found that the TCR thresholds were similar in αβ and γδ T cells *in vitro* (Fig. 4B). The higher membrane CD3 expression of γδ T cells under no or low stimulation suggested an enhanced basal TCR strength in these cells (Fig. 4C). The enhanced TCR signaling is confirmed by increased level of ERK1/2 phosphorylation in γδ T cells at the resting state (Fig. 4D). Thus, our data supports the model that the increased intracellular cholesterol content favors lipid raft formation and enhanced TCR signaling strength in γδ T cells. Our data also provide direct evidence linking high intracellular cholesterol level to the activation of γδ T cells. MβCD-mediated cholesterol depletion lowers the lipid raft content of γδ to the same level as αβ T cells, and significantly attenuated the expression of activation markers in γδ T cells (Fig. 3C–E). On the other hand, increasing cellular cholesterol enhanced the activation status of αβ T cells (Fig. 3F). The cholesterol depletion and loading studies support the notion that cellular cholesterol content directly and positively impacts T cell activation.

Our data provides novel evidence that elevated cellular cholesterol content contributes to rapid proliferation and activation of γδ T cells, which are well-recognized hallmarks of these cells [35]. However, the mechanism by which cholesterol accumulates in γδ T cells requires further investigation. More studies are underway to explore what factors/signaling pathways are involved in this process. γδ T cells are potent producers of effector cytokines such as IL-17 and IFNγ [39]. The fate of IL-17- or IFNγ- producing subsets is pre-determined by CD27 and other factors such as TGFβ in the thymus [36], [40], [41]. The two subsets are differentially regulated and respond to different stimuli [42], [43]. For example, antigen-naïve γδ T cells predominantly produce IL-17 for neutrophil recruitment and resolution of early infection. On the other hand, antigen-experienced γδ T cells produce IFNγ at the site of infection [44]. Particularly, γδ T cells are found to be the major source of IL-17 in naïve mice, and IL-17 production by γδ cells can be further modulated in various pathological conditions including atherosclerosis [36], [40], [43], [45], [46]. Although γδ cells have not been directly studied in atherosclerosis, given that atherosclerosis is driven by both cholesterol and T cells, It would be interesting to study whether cholesterol regulates γδ subset determination, and whether γδ T cells play a pivotal role in atherogenesis. For instance, increased cholesterol levels may favor the development of a specific γδ subset or further regulate its responsiveness to stimuli. We plan to investigate whether the development of γδ T cell subsets, as well as the effectiveness and activation of these subsets are affected by elevating cholesterol levels *in vivo*.

In sum, γδ T cells are more proliferative and activated than αβ cells *in vivo*, suggesting γδ cells are at a more “primed for action” status. The increased cholesterol content of γδ T cells suggests that γδ are able to reach the cholesterol checkpoint faster to allow for rapid proliferation and activation [17]. In addition, increased cholesterol levels in the cell resulted in a higher lipid raft content, which promoted γδ T cell activation. Cholesterol depletion experiments confirmed that both lipid raft content and activation markers were perturbed in cholesterol-depleted γδ T cells, thus further proving that intracellular cholesterol levels are key to γδ activation. γδ T cells were shown to have the same TCR threshold as αβ T cells. However, ERK1/2, the key downstream effector of TCR signaling, is heavily phosphorylated in unstimulated γδ T cells, and ERK1/2 inhibition effectively reduced γδ T cell proliferation, suggesting that the “primed for action” phenotype of γδ T cells is the result of enhanced TCR signaling strength due to increased cholesterol content. Taken together, our data provide a novel mechanism by which cholesterol differentially regulates the physiology of αβ and γδ T cell subsets.
